# Rectus Femoris Tendon Autograft in Anterior Cruciate Ligament Reconstruction: Narrative Review

**DOI:** 10.3390/jcm15186935

**Published:** 2026-09-08

**Authors:** Matic Kolar, Goran Vrgoč, David Martinčič

**Affiliations:** 1 Department of Orthopaedic Surgery, University Medical Centre Ljubljana, Zaloška cesta 9, 1000 Ljubljana, Sloveniadavid.martincic@kclj.si (D.M.); 2 Chair of Orthopaedics, Faculty of Medicine, University of Ljubljana, Vrazov trg 2, 1000 Ljubljana, Slovenia; 3 Faculty of Kinesiology, University of Zagreb, Horvaćanski zavoj 15, 10000 Zagreb, Croatia; 4 Department of Orthopaedics and Traumatology, Clinical Hospital “Sveti Duh”, Sveti Duh 64, 10000 Zagreb, Croatia

**Keywords:** ACL, reconstruction, graft, rectus femoris, quadriceps

## Abstract

**Background/Objectives**: Graft selection remains one of the main hot topics in anterior cruciate ligament (ACL) reconstruction. Despite significant advances in surgical techniques, graft-related factors continue to influence postoperative stability, graft survival, donor-site morbidity, and return-to-sport outcomes. Hamstring tendon (HT), bone–patellar tendon–bone (BPTB), and quadriceps tendon (QT) autografts currently represent the most widely utilized graft choices. Recently, isolated rectus femoris tendon (RFT) harvesting has emerged as a potential alternative, based on the concept of selective utilization of the superficial component of the QT, while preserving deeper structures of the extensor mechanism. **Methods**: A narrative review of the available literature in accordance with the principles of the Scale for the Assessment of Narrative Review Articles (SANRA) on RFT autografts for ACL reconstruction was performed, focusing on anatomical, biomechanical, technical, and clinical studies. Relevant evidence regarding graft morphology, harvesting techniques, biomechanical properties, and clinical outcomes in both primary and revision procedures was synthesized. **Results**: Initial evidence consists of anatomical and biomechanical investigations, as well as three clinical series (one primary, two revision reconstructions). Available data suggest favorable biomechanical characteristics, including adequate graft length, strength, and versatility, together with promising short-term clinical outcomes. **Conclusions**: Isolated RFT autografting represents a promising emerging option for ACL reconstruction. Preclinical and anatomical studies support the rationale for selective harvesting, and the currently available, albeit very limited, clinical data are encouraging. Higher-level prospective evidence is required to define its role in contemporary ACL surgery.

## 1. Introduction

ACL injury is a common injury among physically active individuals that predisposes patients to meniscal injury, chondral degeneration, and early osteoarthritis [1,2]. Consequently, ACL reconstruction has become one of the most frequently performed procedures in orthopedic sports surgery.

Despite advances in surgical techniques and rehabilitation, graft selection remains one of the key considerations in ACL reconstruction. An ideal graft should provide sufficient biomechanical strength, reliable biological incorporation, low donor-site morbidity, reproducible harvesting, and favorable long-term clinical outcomes [3,4].

Bone–patellar tendon–bone (BPTB) and hamstring tendon (HT) autografts have traditionally been the most commonly used grafts, each with well-recognized advantages and limitations [5,6]. More recently, quadriceps tendon (QT) autografts have emerged as an attractive alternative because of their predictable graft diameter, favorable biomechanical properties, and clinical outcomes comparable to HT and BPTB grafts. Advantages of QT grafts include predictable diameter, robust tissue quality, lower rates of anterior knee pain compared with BPTB grafts, and preservation of HT function [7,8].

Although QT grafts have gained substantial popularity only over the last two decades, this technique is not new. The description of QT autografts for ACL reconstruction dates back to Marshall et al. in 1979, who first introduced quadriceps-based reconstruction technique in modern ACL surgery. Initially, the technique failed to gain widespread popularity and was limited to selected cases by many surgeons such as revisions [9]. Subsequently, Fulkerson and Langeland reintroduced the QT concept in 1995 by describing the use of a central QT graft as an alternative to conventional graft options. The authors emphasized several advantages, including substantial graft volume and favorable tissue quality, while noting the difficulties regarding harvesting and related importance for the appropriate technique to avoid complications, especially entering the suprapatellar pouch and losing knee extension [10]. Their work represented an important transitional step between early quadriceps-based reconstructions and contemporary methods.

**Figure 1:**
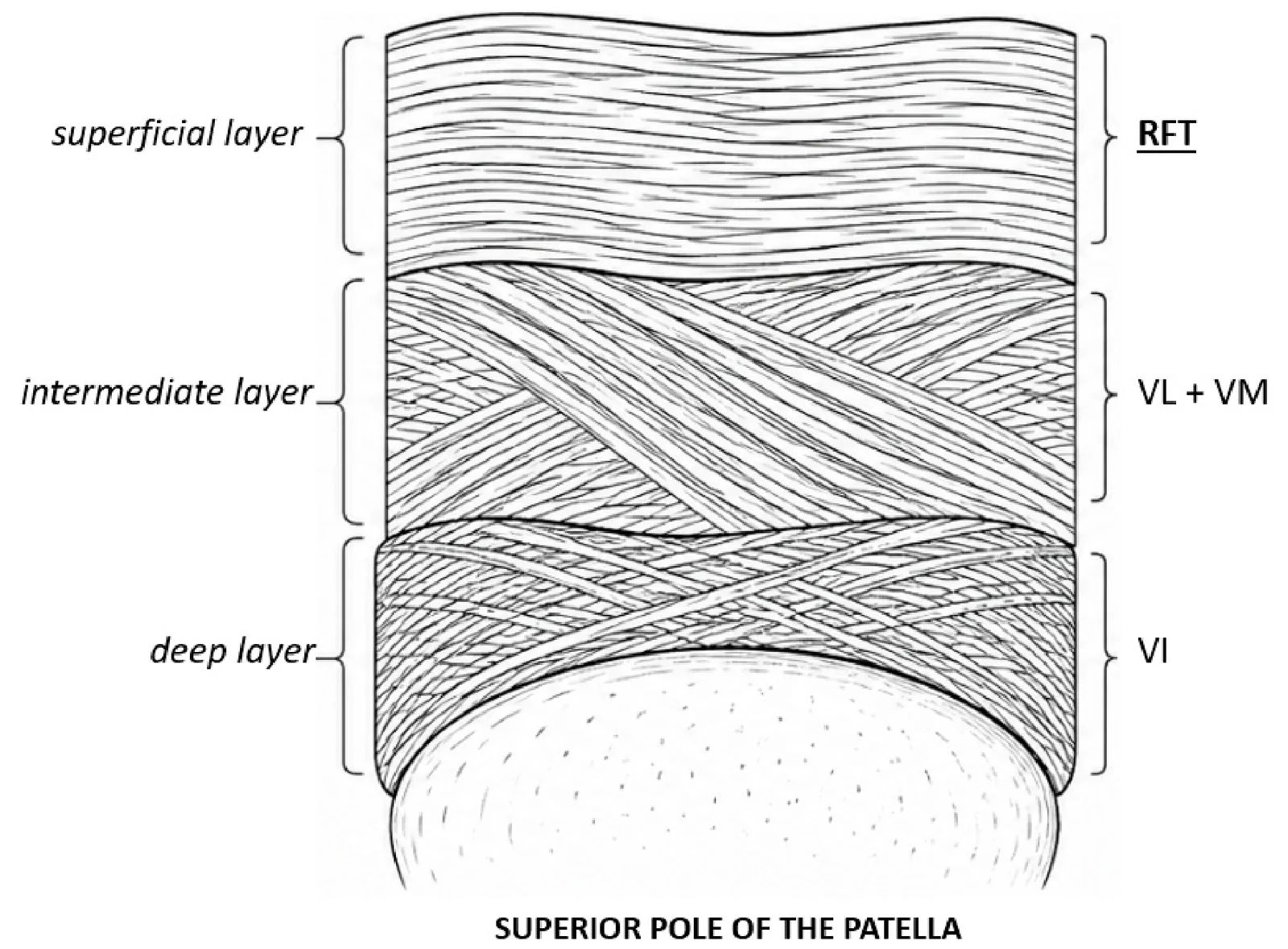
Schematic representation of the QT layers.

The recent interest revival in QT is related to improved understanding of its anatomy. Increasing anatomical knowledge demonstrated that the QT should not be considered a homogeneous structure but rather a complex multilayered construct with potentially distinct biomechanical contributions from individual components. Based on anatomical data, the tendon consists of contributions from the rectus femoris, vastus intermedius (VI), vastus medialis (VM), and vastus lateralis (VL) muscles, with varying fiber orientation and insertion patterns. The superficial layer predominantly comprises rectus femoris fibers with a relatively longitudinal arrangement, whereas deeper layers demonstrate increasingly oblique and interwoven architecture. The intermediate layer is derived from VM and VL, while VI forms the deepest layer [11,12]. A schematic representation of the QT layers is shown in Figure 1.

The isolated RFT graft was established on these anatomical relations. Rather than harvesting a full-thickness QT graft, selective harvesting of the superficial rectus femoris aims to preserve deeper tendon layers and minimize disruption of the extensor mechanism. This concept theoretically combines the advantages of QT grafts, with potentially reduced harvest-related morbidity. Clinically applicable and minimally invasive harvesting techniques focusing on identifying anatomical landmarks and achieving safe and reproducible tendon layer separation were subsequently described by Raman et al., Thamrongskulsiri et al., and Quyen et al. [13,14,15]. Furthermore, the substantial length of the superficial RFT potentially allows multiple-fold graft preparation and combined reconstructive procedures. Recent technical reports described the potential use of a single superficial RFT graft for simultaneous ACL and anterolateral reconstructions, potentially avoiding additional graft harvesting while maintaining sufficient graft dimensions for both intra-articular and extra-articular stabilization procedures [16].

The aim of this manuscript was to provide a narrative review of the currently available evidence regarding isolated RFT autografts for ACL reconstruction.

## 2. Materials and Methods

This study was designed as a narrative review of the currently available evidence re-garding the use of isolated RFT autografts in ACL reconstruction. The objective was to synthesize the anatomical and biomechanical properties, surgical techniques, and clinical results of this novel graft option in both primary and revision reconstructions.

A narrative literature review was performed in accordance with the principles of the Scale for the Assessment of Narrative Review Articles (SANRA) to ensure a transparent and structured literature search [17]. A systematic search of the PubMed database was performed on 1 May 2026, covering all articles available from database inception to the date of the search, using combinations of the following search terms: “rectus femoris tendon”, “superficial quadriceps tendon”, “anterior cruciate ligament reconstruction” and “autograft”. Additional relevant studies were identified through manual screening of reference lists. Studies published in English that evaluated the anatomy, biomechanics, surgical technique, or clinical outcomes of isolated rectus femoris tendon autografts for ACL reconstruction were considered eligible. Technical notes, cadaveric studies, biomechanical investigations, and clinical studies were included because of the limited available literature on this emerging technique. Titles and abstracts were initially screened for eligibility, followed by full-text assessment of potentially relevant articles. Due to the heterogeneity of the available evidence and the limited number of studies, a quantitative synthesis was not performed.

## 3. Results

The literature review identified 12 publications focusing on isolated RFT autografts in ACL reconstruction. These included two anatomical [11,12] and three biomechanical studies [18,19,20], four technical notes and surgical techniques [13,14,15,16], including combined ACL and anterolateral reconstructions [16], and three clinical series [21,22,23]. Among the clinical studies, one evaluated outcomes of primary ACL reconstruction [21], whereas two focused on revision reconstruction procedures [22,23].

### 3.1. Preclinical Evidence

Preclinical evidence regarding isolated RFT grafting remains limited but progressively encouraging. As contemporary ACL surgery increasingly focuses on complete restoration of both anterior and rotational stability, combined procedures involving anterolateral ligament (ALL) reconstruction and lateral extra-articular tenodesis (LET) have become more frequently utilized. In this setting, graft length and configurational versatility represent especially important characteristics.

Although biomechanical investigations of isolated RFT grafts remain limited, general evidence of QT anatomy and biomechanics supports its role in ligament reconstruction. Chivot et al. demonstrated that the mechanical properties of the superficial and deep layers of the central and medial QT are comparable or superior to HT and iliotibial band (ITB), while inferior to the patellar tendon (PT) [18]. Additional support originates from studies of native rectus femoris morphology and tendon properties. Zhu et al. reported that the RFT exhibits anatomical and tensile properties comparable to those of the native ACL [19]. Iriuchishima et al. reported that the RFT has a mean length of approximately 273 mm and a minimum width of 15.3 mm. In contrast, conventional full-thickness QT harvests commonly yield shorter grafts ranging around 70 to 110 mm. Because the RFT may be harvested before merging with deeper tendon layers, substantially longer grafts can be obtained, allowing preparation of folded graft configurations and combined reconstructive procedures [11,24].

The most in-depth biomechanical investigation so far was performed by Mestriner et al., who evaluated isolated RFT graft properties using fresh-frozen cadaveric specimens. The authors compared four constructs of double rectus femoris (DRF), single rectus femoris (SRF), PT, and ITB grafts. Double RFT constructs demonstrated ultimate load-to-failure values of approximately 1978 ± 338 N compared with 1824 ± 557 N for PT grafts, without statistically significant differences. Furthermore, single RFT constructs exhibited biomechanical properties comparable to ITB commonly used in anterolateral procedures. These findings support isolated ACL reconstruction, as well as combined procedures with anterolateral augmentation using a single graft source [20].

Nevertheless, current biomechanical investigations represent time-zero analyses and do not account for biological incorporation, ligamentization, graft remodeling, tunnel widening, or postoperative functional outcomes. Therefore, although current preclinical findings establish anatomical feasibility and provide a promising biomechanical rationale, clinical evidence remains crucial to validate these observations and determine their relevance in real-world ACL reconstruction outcomes.

### 3.2. Clinical Evidence

#### 3.2.1. Primary Reconstruction

Literature regarding isolated RFT use in ACL reconstruction is scarce, with currently available evidence consisting of a single clinical case series and several technical reports. To date, no comparative prospective studies or randomized investigations evaluating isolated RFT grafting in ACL reconstruction have been published.

Rêgo et al. reported outcomes of the first 80 patients undergoing primary ACL reconstruction using a double-folded isolated RFT autograft. The study cohort consisted of 53% male patients with a mean age of 28.95 ± 8.04 years and a mean follow-up of 18.03 ± 6.94 months. At the final follow-up, patients demonstrated excellent functional outcomes, with a mean postoperative Lysholm score of 97.08 ± 2.63 and 100% of patients achieving the patient acceptable symptom state (PASS) threshold. Reported overall complication rate was 6.25%. Complications consisted of four harvest-site hematomas (5%) and one postoperative infection (1.25%). Importantly, no graft failures were observed at the final follow-up [21].

The authors additionally reported three instances of graft amputation during harvesting (3/83, 3.61%), all of which occurred during the first ten procedures. This may suggest a potential learning curve associated with the technique [21].

Another clinically relevant finding was the reported graft length of approximately 30 cm, allowing preparation of multiple-fold constructs with the potential for revision surgery and combined ligament reconstruction procedures [21].

#### 3.2.2. Revision Reconstruction

Revision ACL reconstruction may represent one of the most attractive indications for RFT utilization, because graft selection in revision settings is commonly limited by previous graft harvesting, tunnel enlargement, and the need for larger graft dimensions. The substantial length and customizable configuration of the isolated RFT may therefore provide particular advantages in these complex situations.

Huber et al. initially described the surgical technique and early clinical results of isolated RFT use in revision ACL reconstruction in a retrospective cohort of 28 patients with a mean follow-up of 41.7 months. The harvested tendon demonstrated favorable dimensions, with graft lengths ranging from 30 to 35 cm and sufficient thickness for quadrupled graft preparation. After a quadrupled construction the mean diameter was 9.2 mm at the tibial and 9.0 mm at the femoral end. Overall seven (25%) patients required revision surgery: two (7.1%) graft tears, two menisceal lesions (7.1%), two extension deficits due to the Cyclops syndrome detected on MRI (7.1%), and one (3.6%) misplaced femoral cortical button. Only one patient (3.6%) complained of prolonged pain at the donor site without detected structural changes or complications upon ultrasound evaluation. Postoperative assessment demonstrated satisfactory knee stability and joint function [22].

Subsequently, Huber et al. performed a retrospective comparative study evaluating isolated RFT and HT autografts in revision ACL reconstruction. 55 patients were included, 28 patients in the RFT and 27 patients in the HT group. Mean follow-up was 40.3 months in the RFT cohort and 61.2 months in the HT cohort. Apart from the graft harvesting technique, surgical procedures were performed similarly between groups. At the final follow-up, no significant differences were observed in patient-reported outcome measures between RFT and HT grafts. Mean IKDC scores were 74.7 ± 10.9 and 74.9 ± 12.9, respectively, while mean Lysholm scores reached 90.9 ± 15.0 and 89.0 ± 14.6. Similarly, postoperative activity levels according to the Tegner scale remained comparable, with a median value of 5 in both groups. Postoperative knee stability assessed by the Lachman test improved significantly in both groups. Re-rupture rates were also comparable, occurring in two patients in each cohort (7.1% in the RFT group versus 7.4% in the HT group) [23].

An additional finding of potential clinical relevance involved graft dimensions and surgical planning. The mean femoral tunnel diameter was larger in the RFT group compared with the HT group (9.0 mm vs. 8.2 mm). Consequently, the use of isolated RFT demonstrated a trend toward reducing the need for two-stage revision procedures by approximately 50% compared with HT grafts, although this difference did not reach statistical significance (*p* = 0.11) [23].

These findings suggest that isolated RFT grafting may represent a valuable addition to the available autograft and allograft options in revision ACL surgery.

## 4. Discussion

The present narrative review summarizes currently available evidence regarding isolated RFT autografts for ACL reconstruction and suggests that this technique may represent a promising evolution of contemporary QT-based reconstruction concepts. Although clinical evidence is extremely limited, currently available anatomical, biomechanical, and early clinical findings collectively support the feasibility of isolated RFT harvesting and its potential role within both primary and revision reconstruction settings.

The anatomical rationale for isolated RFT utilization appears particularly compelling. As the RFT forms the superficial layer of the QT, selective harvesting allows preservation of deeper tendon structures and theoretically reduces disruption of the extensor mechanism while maintaining favorable graft dimensions and tissue characteristics. Biomechanical studies additionally support this concept, demonstrating sufficient tensile properties and load-to-failure values comparable to established graft constructs. Furthermore, the substantial graft length reported by Iriuchishima et al. and noted in available clinical studies may provide a unique advantage over conventional QT grafts, particularly in revision procedures and combined ligament reconstruction techniques [11,12,18,19,20,21,22,23,24].

Current clinical evidence remains scarce and consists of one primary and two revision reconstruction studies [21,22,23]. Nevertheless, early findings appear encouraging, as Rêgo et al. reported excellent postoperative functional outcomes and no reported graft failures at short-term follow-ups [21]. Similarly, Huber et al. demonstrated outcomes comparable to HT grafts in revision reconstructions while additionally reporting favorable graft dimensions and related tendency toward reduced requirements for staged revision procedures [22,23].

Several potential advantages of isolated RFT harvesting emerged from the currently available evidence, including preservation of the deeper QT layers, avoidance of HT donor-site morbidity, minimally invasive harvesting, and versatility for different graft configurations and reconstructive procedures [11,12,13,14,15,16,17,18,19,20,21,22,23,24]. Particularly in the context of increasingly individualized reconstruction strategies and combined procedures, graft versatility may represent a crucial advantage. Importantly, successful ACL reconstruction depends not only on graft selection but also on appropriate management of concomitant intra-articular pathology and restoration of rotational stability. Meniscal debridement or repair, treatment of chondral lesions, and, where indicated, associated procedures such as ALL reconstruction or LET represent integral components of contemporary individualized ACL surgery and should be considered alongside graft choice when optimizing clinical outcomes. Although preservation of the deeper QT layers may theoretically reduce donor-site morbidity, potential procedure-specific disadvantages should also be considered. These include rectus femoris retraction, localized quadriceps weakness, muscle atrophy, persistent extension deficits, and possible impairment of explosive quadriceps function, which may influence return-to-sport performance. At present, these potential consequences have not been systematically evaluated, and their incidence and clinical relevance remain to be established in prospective clinical studies.

Importantly, Rêgo et al. reported three instances of graft amputation during harvesting, all occurring within the first 10 procedures [21]. This finding may reflect a potential learning curve associated with the technique. Given the considerable graft length and harvesting characteristics of the RFT, awareness of this potential complication remains important, particularly during early experience with the technique. Careful anatomical identification and controlled harvesting may therefore be essential to minimize technical complications.

Several limitations of isolated RFT grafting should also be acknowledged. Existing clinical evidence is limited without any higher level studies and long-term results. To date, no prospective randomized controlled trials have been published, and the existing clinical literature consists exclusively of retrospective case series with relatively short follow-ups, thereby limiting the strength and generalizability of the available evidence. Consequently, the long-term graft survival, clinical outcomes, and risk of failure remain unknown. Moreover, although biomechanical studies have demonstrated favorable time-zero mechanical properties of isolated RFT grafts, these findings are based on cadaveric models and do not account for biological processes such as graft healing, tunnel incorporation, or ligamentization. Therefore, favorable laboratory results should not be directly extrapolated to long-term clinical performance. In addition, donor-site morbidity and quadriceps strength recovery have not yet been fully investigated. Moreover, the currently available short-term evidence may underestimate procedure-related complications that could become more apparent with longer follow-ups and larger patient cohorts. Potential concerns include persistent extension deficit, arthrogenic muscle inhibition, anterior knee pain, and patellofemoral symptoms or osteoarthritis, particularly when graft harvesting is technically suboptimal. These potential risks require further evaluation in prospective studies with comprehensive functional assessment and long-term follow-up. Finally, the observed learning curve may represent an additional consideration during early implementation. However, the currently available evidence suggests that isolated RFT grafting could be regarded as a promising graft option for primary and revision ACL reconstruction, as well as for additional stabilization procedures, while awaiting further prospective comparative studies with long-term follow-up.

## 5. Conclusions

Isolated RFT autografting represents a promising emerging option for ACL reconstruction. Preclinical and anatomical studies support the rationale for selective harvesting with favorable graft characteristics. The surgical technique appears reproducible and applicable in both primary and revision settings. However, currently available literature remains very limited; therefore, additional clinical data, particularly multicenter prospective comparative studies and higher-level evidence, are required to further define the role of RFT in contemporary ACL surgery.

## Data Availability

No new data were created or analyzed in this study. Data sharing is not applicable to this article.

## Abbreviations

The following abbreviations are used in this manuscript:

ACL	Anterior cruciate ligament
HT	Hamstring tendon
BPTB	Bone–patellar tendon–bone
QT	Quadriceps tendon
RFT	Rectus femoris tendon
VI	Vastus intermedius
VM	Vastus medialis
VL	Vastus lateralis
ALL	Anterolateral ligament
LET	Lateral axtra-articular tenodesis
DRF	Double rectus femoris
SRF	Single rectus femoris
PT	Patellar tendon
ITB	Iliotibial band
